# Supplementary material for: Rich nature of Van Hove singularities in Kagome superconductor CsV3Sb5
Source: Nat Commun. 2022 Apr 25;13:2220. doi: 10.1038/s41467-022-29828-x (PMC9038924; doi:10.1038/s41467-022-29828-x)
Supplement: Supplementary file 1 — Supplemental Information [file 41467_2022_29828_MOESM1_ESM.pdf]

Supplementary Materials

Rich Nature of Van Hove Singularities in Kagome Superconductor CsV_3Sb_5

Yong Hu[1,#,*], Xianxin Wu[2,3,#], Brenden R. Ortiz[4,#], Sailong Ju[1], Xinloong Han[5,6], Junzhang Ma[7,8,9], Nicholas C. Plumb[1], Milan Radovic[1], Ronny Thomale[10,11], S. D. Wilson[4], Andreas P. Schnyder[2,*], and Ming Shi[1,*]

[1]Swiss Light Source, Paul Scherrer Institut, CH-5232 Villigen PSI, Switzerland
[2]Max-Planck-Institut für Festkörperforschung, Heisenbergstrasse 1, D-70569 Stuttgart, Germany
[3]CAS Key Laboratory of Theoretical Physics, Institute of Theoretical Physics, Chinese Academy of Sciences, Beijing 100190, China
[4]Materials Department and California Nanosystems Institute, University of California Santa Barbara, Santa Barbara, California 93106, USA
[5]Department of Physics and Center of Theoretical and Computational Physics, University of Hong Kong, Hong Kong, China.
[6]Kavli Institute of Theoretical Sciences, University of Chinese Academy of Sciences, Beijing, 100049, China
[7]Department of Physics, City University of Hong Kong, Kowloon, Hong Kong, China
[8]City University of Hong Kong Shenzhen Research Institute, Shenzhen, China
[9]Hong Kong Institute for Advanced Study, City University of Hong Kong, Kowloon, Hong Kong, China
[10]Institute for Theoretical Physics, University of Würzburg, Am Hubland, D-97074 Würzburg, Germany
[11]Department of Physics and Quantum Centers in Diamond and Emerging Materials (QuCenDiEM) group, Indian Institute of Technology Madras, Chennai 600036, India

#These authors contributed equally to this work.

*To whom correspondence should be addressed:

Y.H. (yonghphysics@gmail.com), A.P.S. (a.schnyder@fkf.mpg.de), M.S. (ming.shi@psi.ch).

Contents

1. Photon energy-dependent measurements on CsV_3Sb_5

2. Van Hove singularities below and above the CDW transition temperature in CsV_3Sb_5

3. Identification of near-E_F van Hove singularities in CsV_3Sb_5

4. Higher-order van Hove singularities in CsV_3Sb_5

5. Photoemission matrix element analysis and orbital character of the kagome bands in CsV_3Sb_5

6. *m*-type van Hove singularities in AV_3Sb_5 (A=Cs, K)

7. theoretical photoemission matrix element analysis for the higher-order VHS band

8. References

1. Photon energy-dependent measurements on CsV_3Sb_5

According to the DFT calculations, the Sb *p*-orbital of CsV_3Sb_5 (band structure along the Γ–A direction) shows more obvious k_z dispersion along the Γ–A direction (Fig. 1b), which can be used to determine the k_z experimentally. Specifically, the electron band with the highest (resp. lowest) band bottom appears at the Γ (resp. A) point. We found that in the CDW phase the electron band around Γ is quite complicated since a double-band feature can be formed. To better visualize the k_z dependence of the electron band at Γ, we performed ARPES measurements at 200 K ($T >> T_{CDW}$) with higher photon energies (Fig. 1a). From this, an overall periodic evolution of the electron band as a function of photon energy (and thus k_z) can be unambiguously identified (Fig. 1c), from which we obtain the value of the inner potential V_0 of CsV_3Sb_5 to be 11.8 *eV*. Thus, the 78 *eV* photons used in the main text correspond to the k_z = 0 plane.

Despite the DFT calculations reveal a relatively obvious k_z dispersion in CsV_3Sb_5 (Fig. 1d), we find that the k_z dispersion in the experiment is much weaker than that in the DFT calculations. As shown in Fig. 1c, compared with the DFT calculations (Fig. R2c), the bandwidth of Sb p_z band along k_z determined in the experiments is ~200 *meV*, which is only about half of that in the calculations (430 *meV*). These results indicate that even for the Sb p_z orbital with weak correlation effect, we need a renormalization factor of ~2 to match experimental and theoretical bandwidth. Regarding the V *d*-orbitals, even in the DFT calculations (Fig. 1d), compared with Sb p_z orbital, the energy bands around M (L) in the calculations exhibit weaker k_z dispersion. We may even need a large renormalization factor considering the stronger correlation effect of 3*d* orbitals. Indeed, we find that the VHS bands generally exhibit weaker k_z dispersion than the DFT calculations (Figs. 1d-f), while the evolution of the VHS1 band, as it crosses from below to above the E_F, is clearly observed. Moreover, the flat dispersion of VHS1 band is consistently seen in different k_z = 0 planes (as indicated by the orange dashed curve and black arrow in Fig. 1e), despite minor differences in spectral intensity. Notably, the k_z evolution of the VHS1 band is consistent with the k_z periodicity determined by Sb k_z orbital around Γ, i.e., 54 *eV*, 78 *eV* and 108 *eV* all correspond to the k_z = 0 plane, unambiguously confirming the estimated inner potential. We note that the flat feature of VHS1 in 78 *eV* (also presented in our main text) is relatively weaker compared to the 54 *eV* and 108 *eV* data (marked as the vertical arrow in Fig. 1e). On the other hand, the intensity of the VHS1 band below E_F in 78 *eV* is much stronger than the corresponding band in the 54 *eV* and 108 *eV* data (highlighted by the red horizontal arrows in Fig.

1e). We attribute this difference to matrix element effects, that depends on electron momentum, and on the energy and polarization of the incoming photon [6]. Regarding the k_z dispersion of VHS2, we did not observe noticeable k_z dispersion of the VHS2, suggesting the k_z dispersion is weaker than the one of the DFT calculations.

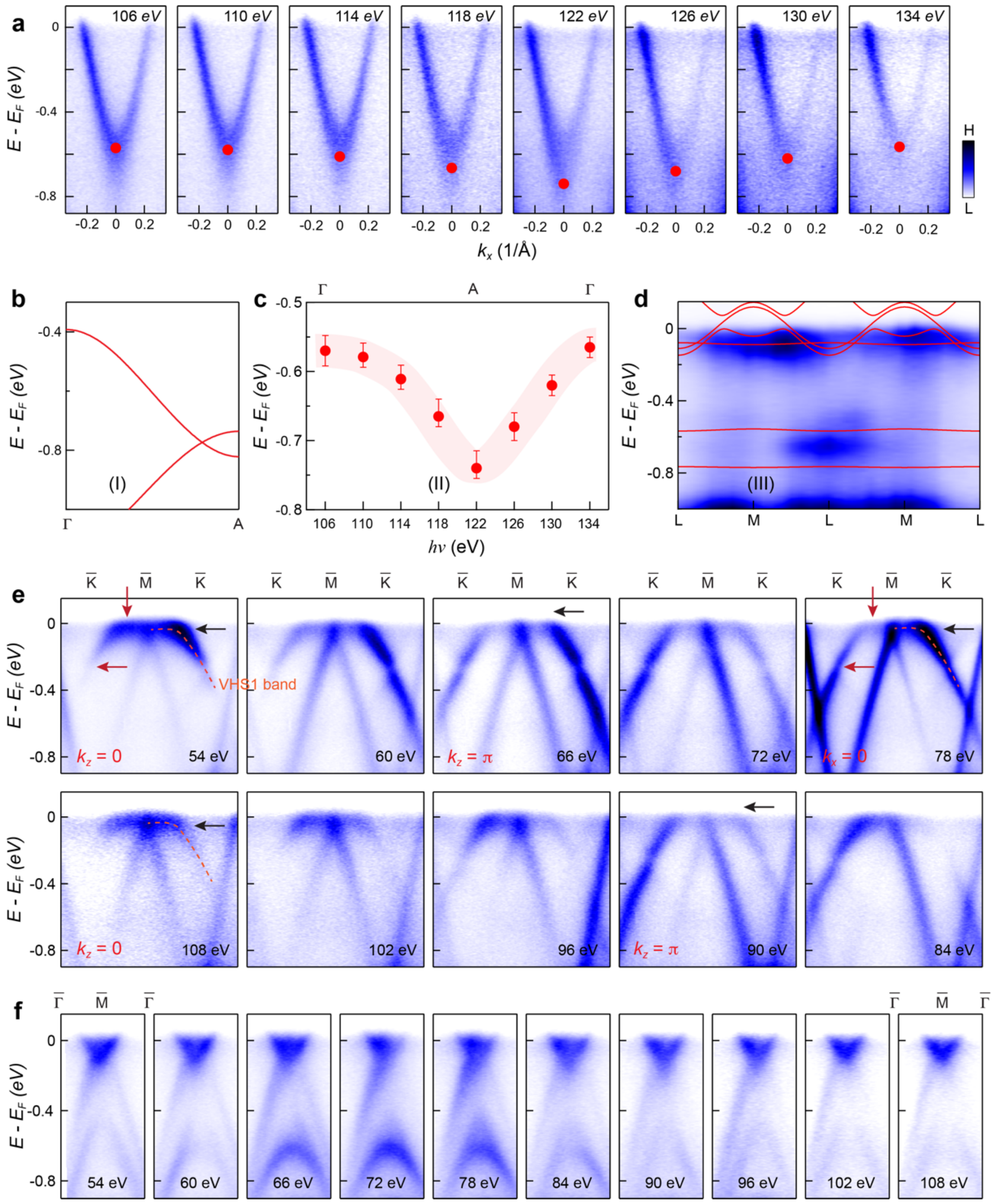

Fig. 1| Photon energy-dependent measurements on CsV_3Sb_5. a The electron-like band as a function of photon energies, measured at 200 K. The red circles mark the evolution of the band bottom. **b** DFT calculated bands along the Γ - A direction. **c** The energy of the band bottom as a function of photon energies, extracted from (a). We note that the energy difference of the band bottom of the electron-like band around Γ(A) is ~200 *meV* in the experiment and ~430 *meV* in the calculations. **d** Photoelectron intensity plot along the ML dispersion. The red lines are the DFT calculations. **e,f** Photon energy-dependent band structures of the VHS bands along the $\bar{K}$ - $\bar{M}$ - $\bar{K}$ direction (e) and $\bar{\Gamma}$ - $\bar{M}$ direction (f). Orange dashed curve is guides to the eye for VHS1 band.

2. Van Hove singularities below and above the CDW transition temperature in CsV_3Sb_5

According to our temperature dependent measurements, the CDW order has some effect on the band dispersion around the M point, except for some peak broadening and an energy shift at the $\bar{M}$ point. Below (20 *K*) and above (130 *K*) the 94 *K* CDW transition temperature (Fig. 2a and 2b, respectively), the peak position of the energy distribution curves (EDCs) taken at the flat feature of VHS1 is almost unchanged [Fig. 2c(i)], while the peak position of the EDCs around $\bar{M}$ is strongly temperature-dependent [Fig. 2c(ii)]. It is important to note that in the region around $\bar{M}$, despite the fact that VHS1 and VHS2 contribute the band dispersion near E_F, the energy position of the VHS1 hardly changes with temperature [Fig. 2c(i)]. Thus, the peak broadening and shift [Fig. 2c(ii)] at low temperature (20 *K*) are related to VHS2, which could be closely associated with the band renormalization driven by the CDW [7].

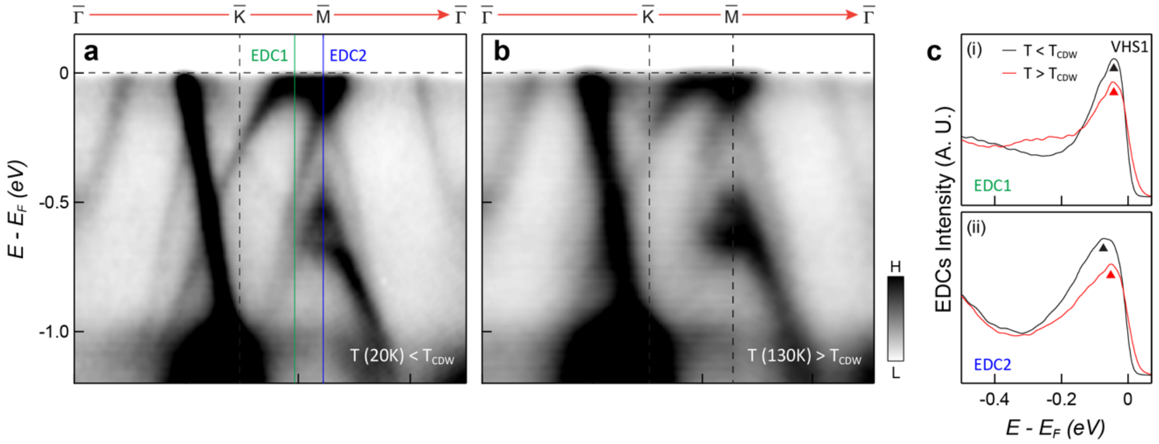

Fig. 2| Van Hove singularities below and above the CDW transition temperature. a Experimental band structure along the $\bar{\Gamma}$ - $\bar{K}$ - $\bar{M}$ - $\bar{\Gamma}$ path, measured with 90 eV, at 20 *K*. **b** Same as (a), but obtained at 130 *K*. **c** Temperature evolution of the EDCs taken at the flat feature of VHS1 (ii) and at the $\bar{M}$ point (ii). The positions of the EDCs are indicated by the green and blue line in (a), respectively. The triangle below the peak indicates the peak position.

3. Identification of near-E_F van Hove singularities in CsV_3Sb_5

Figures 3a and 3b show the second derivative images [8] of the original data presented in Fig. 2c in the main text. The VHS1 with a flat dispersion along $\bar{M}$ - $\bar{K}$ can be clearly seen in Fig. 3a. Taking the second derivative of the band structure (Fig. 2c) with respect to the momentum can well quench the flat feature of VHS1, thereby clearly visualizing the VHS2 (Fig. 3b). To reveal the upwards-curving dispersion along the $\bar{M}$ - $\bar{\Gamma}$ direction, we performed ARPES measurements at 200 *K* (Fig. 3c), and the Fermi-Dirac function was divided out in order to gain information about the thermally populated states slightly above E_F [9]. The upwards-curving dispersion of the near-E_F VHSs is clearly observed, exhibiting the saddle points around $\bar{M}$ point in CsV_3Sb_5.

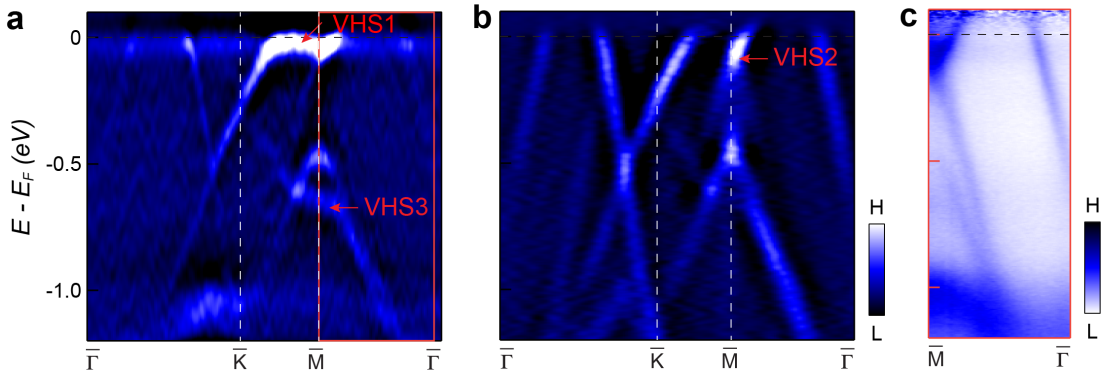

Fig. 3| The near-E_F Van Hove singularities in CsV_3Sb_5. a,b Experimental determined band dispersions along the $\bar{\Gamma}$ - $\bar{K}$ - $\bar{M}$ - $\bar{\Gamma}$ path. The spectra are the second derivative of the original ARPES spectra (shown in Fig. 2c) with respect to the energy (a) and momentum (b), respectively. The blue dashed curves are guides to eye for the VHSs. **c** The ARPES spectrum divided by the Fermi-Dirac function along the $\bar{M}$ - $\bar{\Gamma}$ direction, acquired at 200 *K*. The energy-momentum range is the same as the selected region in (a), as indicated by the orange box. The Fermi-Dirac function is divided out to reveal the energy region slightly above the E_F.

4. Higher-order van Hove singularities in CsV_3Sb_5

In order to study the dispersive nature for the VHS1, we can first write down an effective model around M. With the theory of invariant, the effective Hamiltonian including up to the quartic terms reads,

$$H_M(k) = M + a_1 k_x^2 + a_2 k_x^4 - b_1 k_y^2 - b_2 k_y^4,$$

where k_x and k_y are crystal momenta relative to the M point. With our fitting to the DFT bands of VHS1 near M point, we obtained $a_1 = 4.4, b_1 = 0.6\ \mathrm{eV} \cdot \mathrm{Å}, a_2 = -9.2, b_2 = 13.9\ \mathrm{eV} \cdot \mathrm{Å}^2$ and $M = 0.042$ eV. We find that the effective mass along MK direction is much larger than the MΓ direction, resulting a flat dispersion. The condition for perfect nesting in hexagonal system is $\frac{a_1}{b_1} = 3$. In the DFT fitting, this value is $\frac{a_1}{b_1} = 7.3$, indicating a much less pronounced Fermi surface nesting. Moreover, in the ARPES measurements, the band dispersion of VHS1 along the $\bar{\mathrm{M}}$ - $\bar{\mathrm{K}}$ is even flatter (Figs. 4a-4c) due to the band renormalizations from interactions. According to the fitting of the ARPES data with considering the quadratic term, we obtain $b_1 = 0.06\ \mathrm{eV} \cdot \mathrm{Å}$ and $b_2 = 13.0\ \mathrm{eV} \cdot \mathrm{Å}^2$ (Fig. 4c) and b_2 is three orders of magnitudes larger b_1. Without considering the quadratic term, the fitting can be equally well with $b_2 = 13.42\ \mathrm{eV} \cdot \mathrm{Å}^2$ (Fig. 4c). When the quadratic term, i.e., $b_1 = 0$, the quartic term along the MK direction dominates and the VHS is higher-order, featuring a power-law divergent density of states and exotic instabilities (Fig. 4d). Thus, our experimental fitting clearly suggests that the VHS1 is a higher-order VHS (Fig. 4c).

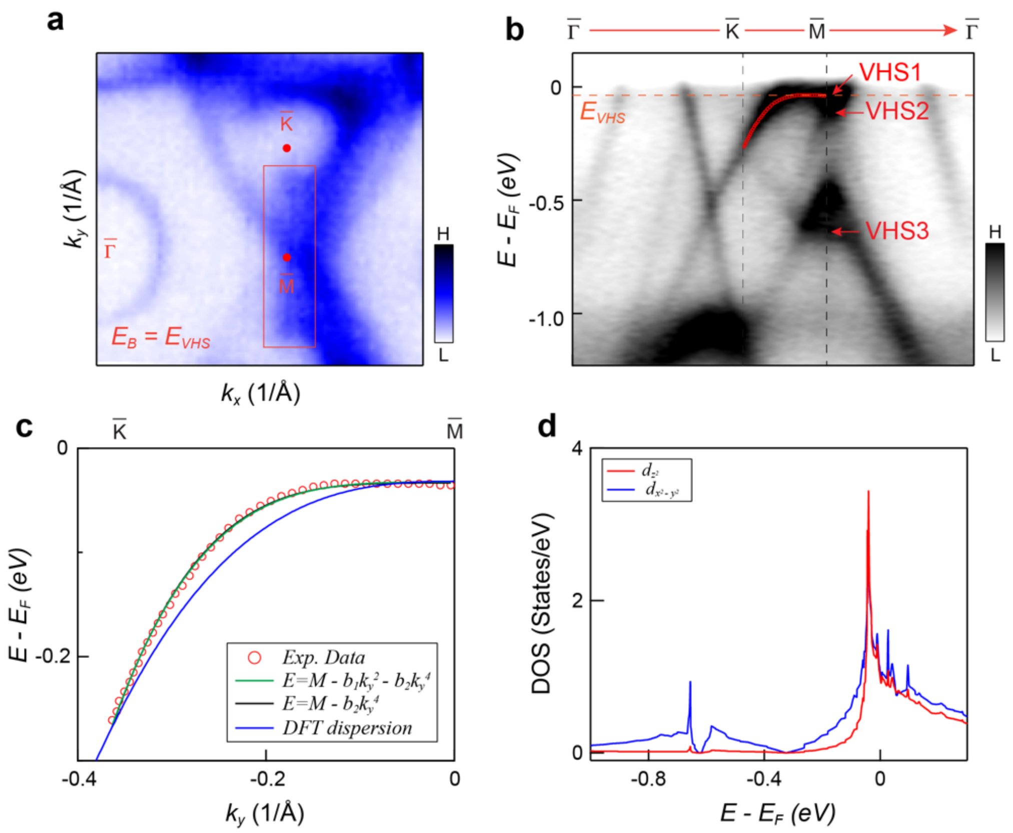

Fig. 4| Quasi high-order Van Hove singularities in CsV_3Sb_5. a Constant energy contour around the Van Hove Singularities (VHS). **b** Experimental band structure along the $\bar{\Gamma}$ - $\bar{K}$ - $\bar{M}$ - $\bar{\Gamma}$ path. The energy distribution curves (EDC)-derived dispersion along the $\bar{M}$ - $\bar{K}$ is shown in red. The orange dashed line marks the energy position (E_{VHS}) of the constant energy contour shown in (a). The red box in (a) and (b) highlights the flat feature along the $\bar{M}$ - $\bar{K}$. **c** Fittings of the measured dispersion along the $\bar{M}$ - $\bar{K}$ by $E = M - b_1 k_y^2 - b_2 k_y^4$ form (the green line) and $E = M - b_2 k_y^4$ form (the black line). The red dots represent the experimental data shown in (b). The momentum position of the $\bar{M}$ is set to 0 for the fittings. Blue curve shows the DFT calculated dispersion. **d** Orbital-resolved Density of states (DOS) from the V kagome sites for the DFT bands in the k_z=0 plane. The peak around -0.05 *eV* from d_{z2} and d_{x2-y2} orbitals is attributed to the higher-order VHS1.

5. Photoemission matrix element analysis and orbital character of the kagome bands in CsV_3Sb_5

In photoemission process, the photoemission intensity measured in an ARPES experiment is proportional to the matrix element term $I_0(\mathbf{k},\nu,\mathbf{A}) \propto |M_{f,i}^{\mathbf{k}}|^2$, where $|M_{f,i}^{\mathbf{k}}|^2 \propto |\langle\phi_f^{\mathbf{k}}|\boldsymbol{\varepsilon}\cdot\boldsymbol{x}|\phi_i^{\mathbf{k}}\rangle|^2$. Here $\boldsymbol{\varepsilon}$ is a unit vector along the polarization direction of the vector potential $\mathbf{A}$, $\phi_f^{\mathbf{k}}$ and $\phi_i^{\mathbf{k}}$ are the final-state wave function and the initial wave function, respectively. Since the final-state wave function $\phi_f^{\mathbf{k}}$ itself must be even [6], the appearance or disappearance of the photoemission signal from a certain orbital depends on the symmetry of $\boldsymbol{\varepsilon}\cdot\boldsymbol{x}|\phi_i^{\mathbf{k}}\rangle$ with respect to a given mirror plane. In order to have nonvanishing photoemission intensity, $\boldsymbol{\varepsilon}\cdot\boldsymbol{x}|\phi_i^{\mathbf{k}}\rangle$ must be even with respect to the mirror plane. Figure S5a illustrates a sketch of spatial symmetries of vanadium (V) $3d$ orbitals. In our experimental data and theoretical calculations, we define the $\bar{\Gamma}$ - $\bar{\mathrm{M}}$ ($\bar{\Gamma}$ - $\bar{\mathrm{K}}$) direction is parallel to the k_x (k_y) direction in the reciprocal space, as indicated in Fig. S5b. According to the above selection rules, we analyze the matrix element effects of V $3d$ orbitals along two high symmetry cuts (horizontal and vertical) under two polarization geometries. The results are summarized in Table I. In the experimental geometry of our polarization-dependent ARPES (Fig. 3a in the main text), the mirror plane is defined by the normal of the sample surface and analyzer slit, which is horizontal to the incident plane. In linear horizontal (*LH*) polarization geometry, the electric field vector $\mathbf{A}$ of the incident light lies within the mirror plan, while in linear vertical (*LV*) polarization geometry, its $\mathbf{A}$ is perpendicular to the mirror plane. When aligning the $\bar{\Gamma}$ - $\bar{\mathrm{M}}$ direction of the sample to along the analyzer slit, d_{xz}, d_{z^2} and $d_{x^2-y^2}$ are all of even symmetry with respect to the mirror plane. Therefore, photoemission signals from these orbitals are detectable in the *LH* geometry. However, d_{yz} and d_{xy} are odd with respect to the mirror plane, and thus their photoemission signals are only allowed in the *LV* geometry. Similarly, we can also analysis the results when the analyzer slit is along the $\bar{\Gamma}$ - $\bar{\mathrm{K}}$ direction (TABLE I).

TABLE I. Summary of the detectable *3d* orbitals under *LH* and *LV* polarization geometries, when the mirror plane is along different high-symmetry directions.

High-symmetry directions	Geometry	V $3d$ orbitals				
$\bar{\Gamma}$ - $\bar{\mathrm{M}}$	LH	d_{xz}			$d_{x^2-y^2}$	d_{z^2}
$\bar{\Gamma}$ - $\bar{\mathrm{M}}$	LV		d_{yz}	d_{xy}		
$\bar{\Gamma}$ - $\bar{\mathrm{K}}$	LH		d_{yz}		$d_{x^2-y^2}$	d_{z^2}
$\bar{\Gamma}$ - $\bar{\mathrm{K}}$	LV	d_{xz}		d_{xy}		

Next, we analysis the matrix element effects of the kagome bands that constitute the VHSs in CsV_3Sb_5 (Figs. 3c-h in the main text and Figs. 5c,5d). For convenience, we label the bands that form the VHS3, VHS2, VHS1 and VHS 4 as α, β, γ, δ, respectively (Figs. 5c and 5d). Considering that the α bands are simultaneously detected along the $\bar{\Gamma}$ - $\bar{K}$ (Fig. 5g) and $\bar{\Gamma}$ - $\bar{M}$ (Fig. 5h) directions in the *LV* geometry, the α bands are contributed by d_{xy} orbital. Likewise, we can determine that the β bands are dominated by d_{yz} orbital, the γ bands should be contributed by $d_{x^2-y^2}$/ d_{z^2} orbitals, while the δ bands should originate from d_{xz} orbital.

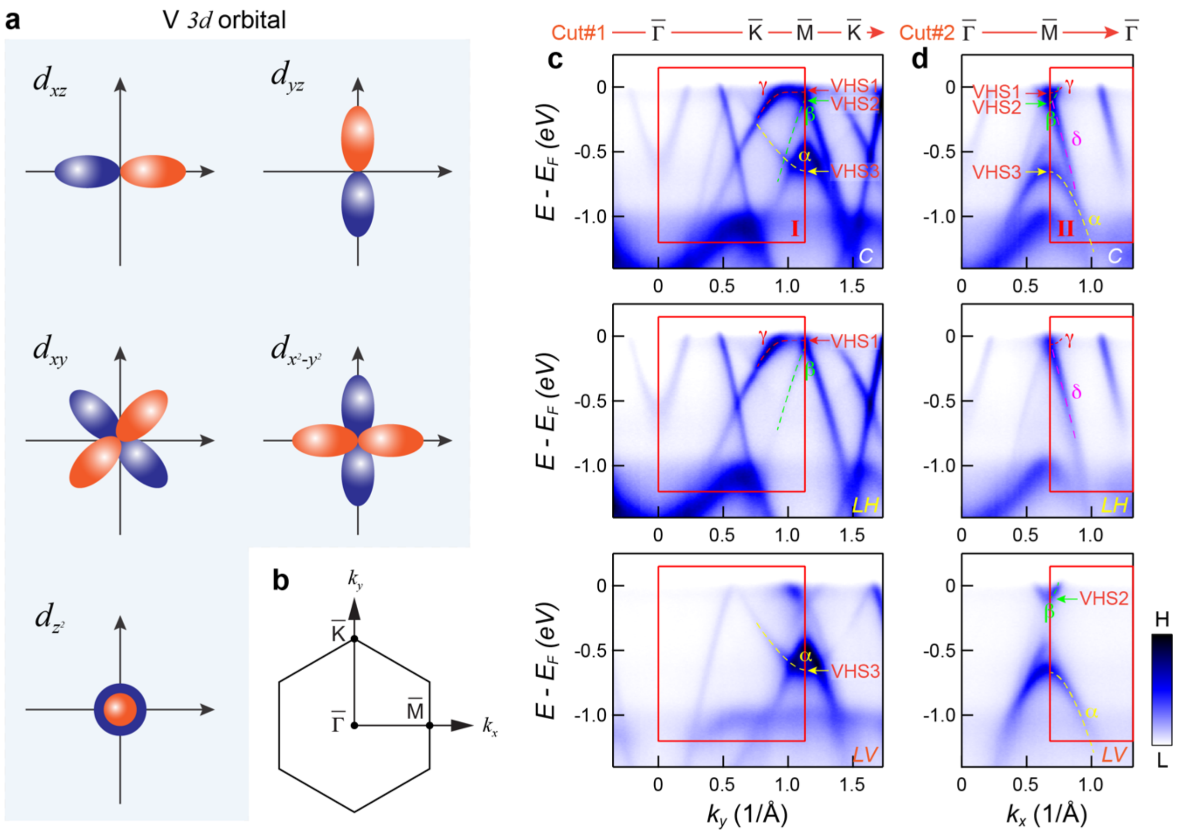

Fig. 5| Determination of the orbital nature of the kagome bands in CsV_3Sb_5. a The spatial symmetries of vanadium (V) *3d* orbitals. **b** The definition of the coordinate system in the reciprocal space. **c,d** Band dispersion the along the $\bar{\Gamma}$ - $\bar{K}$ - $\bar{M}$ - $\bar{K}$ [Cut#1, (c)] and $\bar{\Gamma}$ - $\bar{M}$ - $\bar{\Gamma}$ [Cut#2, (d)] directions, respectively. The momentum directions of the cuts are indicated by the red arrows in (b). The bands are measured with circularly polarized light, at 20 K. **e,f** and **g,h** Same as (c),(d), but probed with linear horizontal (*LH*) (e,f) and linear vertical (*LV*) (g,h) polarizations, respectively. The dispersions in (c-h) are the same as the results shown in Figs. 3(c-h).

6. *m*-type van Hove singularities in AV_3Sb_5 (A=Cs, K)

DFT band structures for CsV_3Sb_5 and KV_3Sb_5 are shown in Fig. 6. In KV_3Sb_5, the *m*-type Van Hove singularities (VHS) above the Fermi level can be clearly identified due its odd-parity at M point (irrep. B_{1u}). In CsV_3Sb_5, the B_{1u} state shift up in energy and goes above B_{3g} state, inducing an anti-crossing between the two bands along MG direction. Therefore, the dispersive d_{xz} bands along the MΓ in CsV_3Sb_5 still belongs to *m*-type VHS.

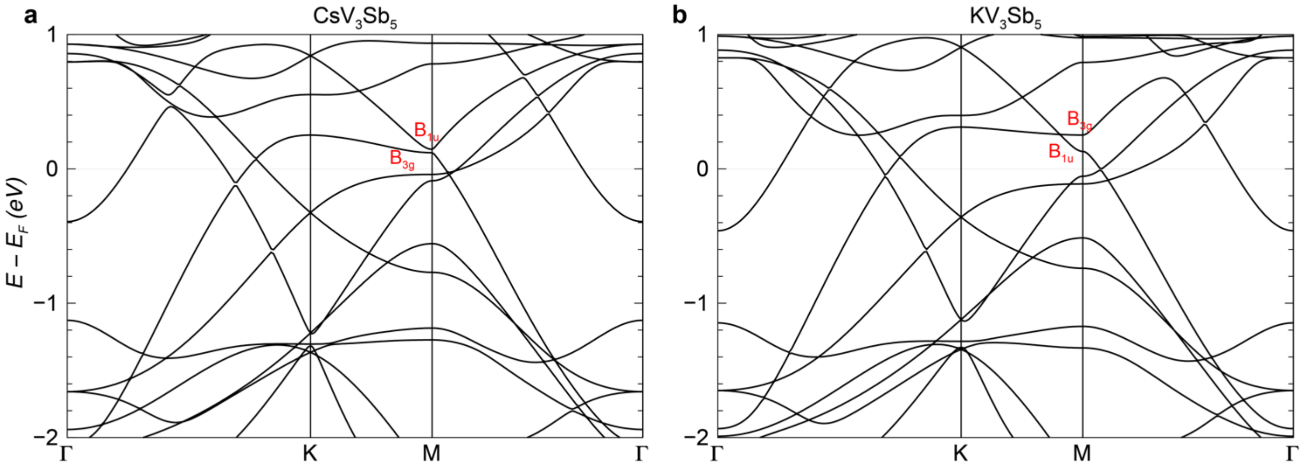

Fig. 6| DFT band structures for CsV_3Sb_5 and KV_3Sb_5.

7. Theoretical photoemission matrix element analysis for the higher-order VHS band at 78 *eV*

Although the flat feature of VHS1 band is clearly shown in Fig. 3c (and Figs. 2c and 3i) in the main text, we notice that the intensity of the flat-top dispersion of VHS1 around the $\bar{\mathrm{M}}$ point is weakened under the *LH* geometry (Fig. 3e). However, the full flat dispersion of the VHS1 can be observed at other photon energies (54 *eV*, 108 *eV*, corresponding to the $k_z = 0$ as same as 78 *eV*, Fig. 7d), indicating that the diminished intensity of the flat-band top at 78 *eV* (Fig. 3e) is attributed to the matrix elements effect. In this section, we will demonstrate that the relatively weak feature of the VHS1 flat band along the M-K at 78 *eV* in the measurements can be attributed to the matrix element effects of dx2-y2 and dz2 orbitals.

Our calculations show that the higher-order VHS band (near the M point) is dominantly contributed by V *d* orbitals. The corresponding wavefunction can be written as: $\phi_i^{\mathbf{k}} = \sum_{\alpha n} \mathrm{b}_{\alpha n}^{\mathbf{k}} W_{\alpha n}(\mathbf{k})$, where $\alpha = A, B, C$ is the sublatttice index, $\mathrm{n} = 1,2,3,4,5$ denotes xz, yz xy, x2-y2 and z2 orbitals and $W_{\alpha n}(\mathbf{k})$ is the Wannier function. The photoemission intensity measured in an ARPES experiment is proportional to $I_0(\mathbf{k}, v, \mathbf{A}) \propto \left|\langle \phi_f^{\mathbf{k}} | \boldsymbol{\varepsilon} \cdot \boldsymbol{x} | \phi_i^{\mathbf{k}} \rangle\right|^2 \delta(E_f - E_i - E_p)$, where $\boldsymbol{\varepsilon}$ is a unit vector along the polarization direction of the vector potential **A** and $\phi_f^{\mathbf{k}}$ and $\phi_i^{\mathbf{k}}$ are the final-state wave function and the initial wave function, respectively. Therefore, the photoemission intensity is,

$$I_0(\mathbf{k}, v, \mathbf{A}) \propto |\sum_{\alpha n} \mathrm{b}_{\alpha n}^{\mathbf{k}} \xi_{\alpha n}^{\varepsilon}|^2 \delta(E_f - E_i - E_p)\,,$$

where $\xi_{\alpha n}^{\varepsilon} = \langle \phi_f^{\mathbf{k}} | \boldsymbol{\varepsilon} \cdot \hat{\mathbf{p}} | W_{\alpha n}(\mathbf{k}) \rangle$ is the dipole transition matrix element for orbital n at sublattice α. Considering the experimental setup in the polarized measurement in Figs. 3e and 3g, the incident plane is yz plane. The $\mathrm{M_{yz}}$ mirror reflection transforms $\hat{\boldsymbol{p}}_x \longrightarrow -\hat{\boldsymbol{p}}_x$, $\hat{\boldsymbol{p}}_{y,z} \longrightarrow -\hat{\boldsymbol{p}}_{y,z}$ and $A/B \longrightarrow \mathrm{B/A}, C \longrightarrow C$. yz, x2-y2 and z2 orbitals are even and xz and xy orbitals are odd under the $\mathrm{M_{yz}}$ mirror reflection. Considering all the above relations, we get the following equations:

n is even orbital, i.e., $n = x^2 - y^2, z^2, yz$

$$\xi_{An}^{x} = -\xi_{Bn}^{x}, \xi_{An}^{y/z} = \xi_{Bn}^{y/z}, \xi_{An}^{x} = 0, \xi_{An}^{y/z} \neq 0$$

n is odd orbital, i.e., $n = xz, xy$

$$\xi_{An}^{x} = \xi_{Bn}^{x}, \xi_{An}^{y/z} = -\xi_{Bn}^{y/z}, \xi_{An}^{x} \neq 0, \xi_{An}^{y/z} = 0.$$

The vector potentials in ARPES measurements with *LH* and *LV* polarization are $\mathrm{A_{LH}} = \mathrm{A}_y\hat{\mathrm{y}} + \mathrm{A}_z\hat{\mathrm{z}}$ and $\mathrm{A_{LV}} = \mathrm{A}_x\hat{x}$, respectively. After some algebra, we obtain the ARPES intensity for *LH* and *LV* polarization:

$$I^{LH}(\mathbf{k}) \propto |\sum_{\alpha,n\in \text{even}} [(A_y\xi^{y}_{An} + A_y\xi^{y}_{An})(b^{\mathbf{k}}_{An} + b^{\mathbf{k}}_{Bn}) + (A_y\xi^{y}_{Cn} + A_y\xi^{y}_{Cn})b^{\mathbf{k}}_{Cn}]$$

$$+ \sum_{\alpha,n\in odd} (A_y\xi^{y}_{An} + A_y\xi^{y}_{An})(b^{\mathbf{k}}_{An} - b^{\mathbf{k}}_{Bn})|^2$$

$$= |\sum_{\alpha,n\in \text{even}} [A_{LH}\xi^{LH}_{An}(b^{\mathbf{k}}_{An} + b^{\mathbf{k}}_{Bn}) + A_{LH}\xi^{LH}_{Cn}b^{\mathbf{k}}_{Cn}] + \sum_{\alpha,n\in odd} A_{LH}\xi^{LH}_{An}(b^{\mathbf{k}}_{An} - b^{\mathbf{k}}_{Bn})|^2,$$

$$I^{LV}(\mathbf{k}) \propto |\sum_{\alpha,n\in \text{even}} A_{LV}\xi^{LV}_{An}(b^{\mathbf{k}}_{An} - b^{\mathbf{k}}_{Bn}) + \sum_{\alpha,n\in odd} [A_{LV}\xi^{LV}_{An}(b^{\mathbf{k}}_{An} + b^{\mathbf{k}}_{Bn}) + A_{LV}\xi^{LV}_{Cn}b^{\mathbf{k}}_{Cn}]|^2$$

According to the polarization-dependent measurements (Figs. 3a-3i in the main text) and the DFT calculations (Fig. 3j in the main text), the higher-order band are mainly attributed to dx2-y2 and dz2 orbitals. The dz2 orbital weight of this band increases monotonically but the weight of dx2-y2 is almost a constant from K to M (Fig. 7a, and Fig. 3j in the main text). Around M point, the orbital weight of dz2 and dx2-y2 are close. Most importantly, due to the *p*-type nature, the flat-top band around M is dominantly attributed to dx2-y2 and dz2 at C sublattice. Moreover, according to our DFT calculations, their coefficients along KM line have the opposite phase, i.e., $b^{\mathbf{k}}_{Cx^2-y^2} \approx -cb^{\mathbf{k}}_{Cz^2}$, and the factor c decreases monotonically from K to M. If we only consider the dominant contribution from dx2-y2 and dz2 orbitals in the ARPES intensity, we can get a simplified formula for the ARPES intensity under *LH* polarization around M point, $I^{LH}(\mathbf{k}) \propto |A_{LH}\xi^{LH}_{Cx^2-y^2}b^{\mathbf{k}}_{Cx^2-y^2} + A_{LH}\xi^{LH}_{Cz^2}b^{\mathbf{k}}_{Cz^2}|^2$. If the dipole transition matrix elements with *LH* polarization for dx2-y2 and dz2 are close, there is a cancelation between contributions from two orbitals, generating a diminished intensity around M. If the transition matrix elements for two orbitals have a large difference, the cancellation will not be prominent. This provides a reasonable explanation for the diminished intensity for the flat-top dispersion under *LH* polarization but the strong intensity under circular polarization. To further verify our scenario, we performed theoretical calculations using the coefficient from DFT calculations to simulate the ARPES intensity under *LH* polarization. By adopting $\xi^{LH}_{\alpha n} = 1\ (n \neq z^2)$ and $\xi^{LH}_{\alpha z^2}/\xi^{LH}_{\alpha n} = 0.75$ in the $I^{LH}(\mathbf{k})$ formula, the simulated spectrum is displayed in Fig. 7b, where the observed diminished intensity in the higher-order band near M can be well reproduced. For the case of circular polarization, the ARPES intensity is $I^{C}(\mathbf{k}) \propto |\sum_{n,\alpha}(A_y\xi^{y}_{\alpha n} + A_z\xi^{z}_{\alpha n} + iA_x\xi^{x}_{\alpha n})b^{\mathbf{k}}_{\alpha n}|^2$. By adopting $A_C = A_y\hat{y} + A_z\hat{z} + iA_x\hat{x}$ with $A_y = A_z = A_x/\sqrt{2}$ and $\xi^{x,y,z}_{\alpha n} = 1\ (n \neq z^2)$ and $\xi^{x,y,z}_{\alpha z^2}/\xi^{x,y,z}_{\alpha n} = 2.6$, the simulated spectrum with circular polarization is displayed in Fig. S7(c), where the intensity is strong near M and weak around K. These are qualitatively consistent with the experiments (Figs. 3c and 3e in the main text). However, the matrix element in ARPES spectrum depends on electron momentum,

and on the energy and polarization of the incoming photon [6]. Our theoretical simulation is for the measurement with a photon energy of 78 *eV*.

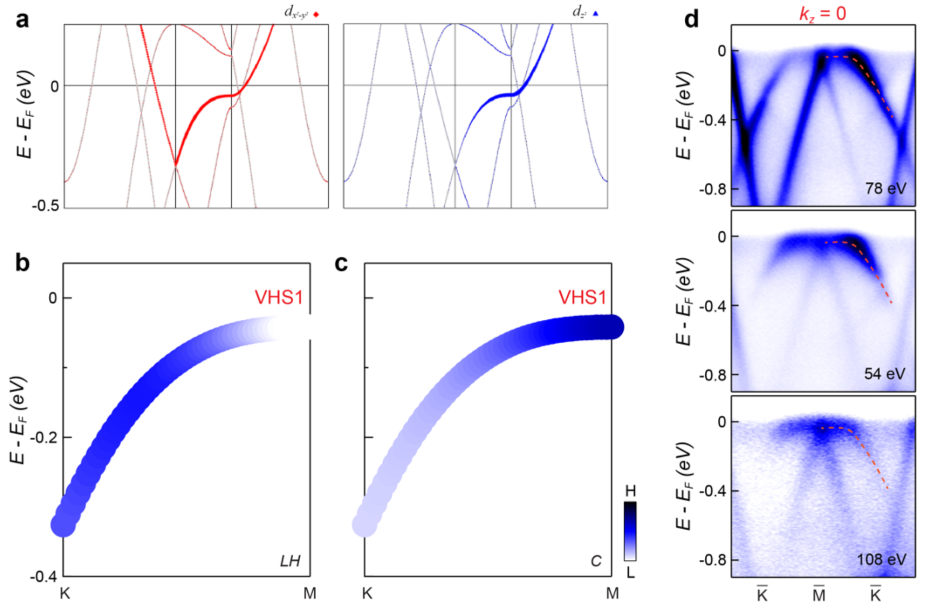

Fig. 7|Photoemission matrix element analysis for the higher-order VHS band. a Orbital character resolved band structure in k_z=0 plane from DFT calculations and the simulated spectrum for the higher-order VHS band (VHS1). **b,c** Simulated ARPES spectrum for the higher-order VHS band (VHS1) under the *LH* polarization (b) and the circular polarization (c). **d** ARPES spectra along the K - M- K direction in different k_z =0 plane, measured with different photon energies.

8. References

[1] Kresse, G. & Hafner, J. Ab initio molecular dynamics for liquid metals, *Phys. Rev. B* **47**, 558–561 (1993).

[2] Kresse, G. & Furthmüller, J. Efficiency of ab-initio total energy calculations for metals and semiconductors using a plane-wave basis set. *Computational Materials Science* **6**, 15–50 (1996).

[3] Kresse, G. & Furthmüller, J. Efficient iterative schemes for ab initio total-energy calculations using a plane-wave basis set. *Phys. Rev. B* **54**, 11169 (1996).

[4] Perdew, J. P., Burke, K. & Ernzerhof, M. Generalized Gradient Approximation Made Simple. *Phys. Rev. Lett.* **77**, 3865 (1996).

[5] Monkhorst, H. J. & Pack, J. D. Special points for Brillouin-zone integrations. *Phys. Rev. B* **13**, 5188 (1976).

[6] Damascelli, A., Hussain, Z. & Shen, Z.-X. Angle-resolved photoemission studies of the cuprate superconductors. *Rev. Mod. Phys.* **75**, 473-541 (2003).

[7] Hu, Y. et al. Topological surface states and flat bands in the kagome superconductor CsV_3Sb_5. *Sci. Bull.* **67**, 495-500 (2022).

[8] Sato, T. et al. Observation of $d_{x2\ -y2}$-like superconducting gap in an electron-doped high-temperature superconductor. *Science* **291**, 1517–1519 (2001).

[9] Greber, T., Kreutz, T. J. & Osterwalder, J. Photoemission above the Fermi Level: The Top of the Minority d Band in Nickel. *Phys. Rev. Lett.* **79**, 4465–4468 (1997).
